# Supplementary material for: Are carbon emissions trading and green financial instruments synergistic? -Comprehensive quantitative research based on content analysis
Source: PLoS One. 2024 Mar 7;19(3):e0298601. doi: 10.1371/journal.pone.0298601 (PMC10919601; doi:10.1371/journal.pone.0298601)
Supplement: S1 Data — (ZIP) [file pone.0298601.s001.zip › Data/地级市Z_F工作报告.zip!/╣π╢1⁄2/╜¡├┼╩╨/└┤╫╘╣1⁄2╓┌║┼í╛┬φ┐╦╩2╛▌═°í┐_2015.docx]

各位代表：

　　我代表市政府向大会作工作报告，请予审议，并请政协委员和列席人员提出意见。

　　2014年工作回顾

　　过去的一年，市政府在省委、省政府和市委的正确领导下，在市人大及其常委会的监督和市政协的支持下，紧抓改革主题和发展主线，继续以“种树、搭桥修路、抓大项目”为抓手统领全局工作，稳增长、调结构、促改革、惠民生等各项举措得到有效落实。

　　一、经济平稳增长

　　全市实现生产总值2083亿元，同比增长7.8%，经济总体实现稳中有进（主要指标见附件1）。江门产业转移工业园连续三年获评省优。先进（装备）制造业增长13.8%。技改投资大幅增长111.7%。诚信企业绿卡计划[1]得到落实，35家企业被认定为骨干企业。海洋经济增加值增长11.5%，完成大广海湾经济区专项规划编制和市、县两级海洋功能区划修编。中微子实验项目启动建设，成立了市技术交易中心和省科技金融综合服务中心江门分中心。金融信贷增长18%，居珠三角第2，成为广东唯一的国家级小微企业信用体系建设试验区，5家企业在境内外上市，为历年数量最多。旅游总收入279亿元，增长25%。现代农业新增8家市级以上龙头企业，新增农民专业合作社340家，在全省率先推动农业“政银保”[2]试点工作。成功举办上海投洽会、光博会、农博会和锦鲤博览会。

　　二、基建稳健推进

　　全市重点项目完成投资500.9亿元，投资完成率达110.6%。江顺大桥完成主跨合龙，江门大道龙舟山隧道实现贯通，凤凰山隧道群、新南路立交、双龙立交、龙湾立交全面铺开。广中江、江罗、新台南延线等高速公路建设进展顺利，中开、鹤开高速完成招标。深茂铁路江门段、江门高新区公共码头项目启动建设。建成李文达大桥、大鳌特大桥、迎宾路立交、新峰路、启超大道、今洲路等一批路桥，完成314公里农村公路建设。全市投入11.2亿元加快宽带网络建设。电网建成110千伏以上线路587公里。全市水利投资完成6亿元，“三防”通讯指挥处置能力提升，新建高标准基本农田34.7万亩，开平和恩平成为中央财政小型农田水利建设重点县。新一轮扶贫“双到”[3]累计筹集帮扶资金2.9亿元。

　　三、改革稳步深化

　　确立了东西两大板块区域协同发展战略，分别成立东部三区一市[4]建设发展协调领导小组和西部台开恩[5]协调发展联席会议制度。市、区财权事权改革和企业投资体制改革扎实推进。创新投融资方式，国资整合成果进一步稳固。商事制度改革扎实推进，新发展市场主体3.7万户、注册资本 177.1亿元，同比增长19.6%、56.8%。全市累计清理压减行政审批事项3155项，压减率达41.6%。整合组建市商务局等5个单位，完成食品药品监管体制改革。农村土地承包经营权确权登记试点工作有序开展。社会治理体系改革取得进展，市本级购买服务项目100个、投入资金4600万元，增长32%，统筹超1.1亿元国有资产开展公益创投活动，新增社会组织916个，培育成立民办社工机构71家。

　　四、环境稳定改善

　　扎实开展绿化大行动，80万人次参加义务植树，植树260万株。全市完成生态景观林带建设48.2公里、碳汇林建设4.56万亩、乡村绿化美化示范村234条，森林覆盖率提高1个百分点，人均公园绿地面积达17.4平方米，成为省林业生态市。潭江流域水环境保护得到加强，完成重点水污染行业和112家禽畜养殖场的综合整治，新建成污水处理厂5座、污水管网161公里，新增污水处理能力6.4万吨/日，全市城镇污水处理率达89.4%。全市城镇生活垃圾无害化处理率达96.3%。加强大气污染防治，完成高污染燃料锅炉整治234台，淘汰“黄标车”[6]及老旧车辆3.31万辆，完成15项火电减排工程。改造提升电机能效功率10.7万千瓦。

　　五、社会民生稳定发展

　　全市各级财政民生投入114.4亿元，增长16.8%，十件民生实事全面完成。顺利通过了国家“创文”复检，长效机制逐步健全。

　　“大民政”建设成效初显，成为全省基本公共服务均等化综合改革试点，底线民生水平明显提升。三区四市城乡居民养老保险基础养老金标准、城乡低保标准、农村五保[7]供养标准、孤儿供养水平、城乡医疗救助水平、低保残疾人生活津贴和重度残疾人护理补贴标准均有提高。建立完善大病保险制度，社会保障面进一步扩大。全市新增养老机构5家、养老床位5031张，居家养老服务网络逐步形成。为2500名残疾人提供“四居”[8]服务。全市共筹慈善捐款1.6亿元。就业形势稳定，全市城镇新增就业4.7万人，失业人员再就业3.2万人，城镇登记失业率2.36%。超额完成省下达的保障房建设任务，解决2794户中低收入、困难群众家庭的住房问题。

　　各项社会事业不断进步。三区四市全部通过国家义务教育基本均衡督导检查，江海、新会成功创建省推进教育现代化先进区，恩平通过省教育强市督导验收，启动了特殊教育学校扩建工程，挂牌成立了社区大学。江门体育中心建设顺利推进，广东银葵综合医院动工建设。有效防控了禽流感、登革热等疫情，人口计生工作完成省下达目标任务。成功举办了世界江门青年大会。文化下乡、进社区工作扎实推进。平安江门建设得到巩固。各项生产安全事故全面大幅下降。外事、港澳、对台、民族、宗教、双拥、优抚、人防、打私、统计、审计、妇女儿童、新闻、出版、档案、方志、气象、红十字会、应急等工作均有新的进展。

　　六、政府建设稳步加强

　　自觉接受人大和政协的监督，办理人大代表建议和政协提案满意率分别达96.3%、100%。认真开展党的群众路线教育实践活动，大力整治“四风”[9]，修改完善和建立相关制度300个。法治政府建设扎实推进。积极发挥市决咨委[10]作用，运用网站、微博、微信等网络问政手段，广集民意、广汇民智。严格贯彻落实中央“八项规定”及领导干部办公用房清理等工作，全市“三公”经费[11]降低18.4%。强化廉政风险防控，坚决查处违法违纪案件。

　　一年来政府工作中的问题主要有：部分经济指标未能完成预期目标任务；产业发展量小质弱；创新动力不强；资源整合不足、利用效率有待提升，环境约束明显；行政管理层次多、效率低、责权不清的现象依然比较突出。对于这些问题，我们将采取更有力措施加以解决。

　　各位代表！过去一年政府工作取得的成绩来之不易。在此，我代表市政府，向关心支持江门发展建设的各界人士表示衷心的感谢！

　　2015年工作安排

　　今年是深化改革的突破年、狠抓落实的决战年，同时是“十二五”规划的收官年，也是“十三五”规划的谋划年。国家“一带一路”[12]和省珠西行动战略为我们带来了新机遇，新常态对我市经济社会转型提出了前所未有的新要求和挑战。今年政府工作的指导思想是：深入贯彻落实党的十八大、十八届三中、四中全会、中央经济工作会议、省委十一届四次全会和市委十二届五次全会精神，适应新常态，谋划新格局，抢抓新机遇，深化“三边”发展战略，推动“东提西进、同城共融”[13]，落实“区域协同、经济蓄势、城市振兴、民生保障、政府提效”五大任务，实现幸福侨乡建设有新的突破。

　　综合各方因素，今年全市经济发展的预期目标为生产总值增长8%左右，一般公共预算收入增长10%（其他指标见附件2）。

　　做好今年的政府工作，关键词就是一个“拼”字，各级各部门要拿出拼搏攻坚、狠抓落实的劲头，做好以下重点工作：

　　一、区域协同

　　东联西拓。对外抓住国家“一带一路”、省打造珠西先进装备制造产业带、建立自贸区等战略机遇，充分发挥我市东联广佛都市圈、深港澳经济圈两大龙头，西扼省战略西拓的区位优势，围绕加快成为珠三角新增长极目标，做好“传”“接”的“中卫”角色。深化与广佛都市圈的对接，推动“江佛一家”，抓好珠中江区域合作和珠西行动计划的落实。着力推进江港澳开展合作，以大广海湾经济区为主要依托，积极创建国家级区域发展合作的重大平台。

　　东提西进。对内按照东部一体、西部协同原则，分东西板块统筹制订发展政策，促进资源优化整合，推动区域内交通一体、产业同兴、环境共治、民生共享，实现同城共融。东部通过江门大道连成一体，推动江门高新区与蓬江江沙示范园、滨江新区联动发展、融合提质，建设先进制造业总部集聚区及智慧产业集群区，打造成为创新型城市先导区，力争纳入珠三角国家自主创新示范区；新会作为大广海湾经济区的先行区，全力整合提升银洲湖产业新城，成为江门先进（装备）制造业主力军；鹤山工业城（共和－鹤城－址山）加紧整合提质，成为江门先进制造业的生力军。西部重点突出交通、旅游、农业、环保协同发展，以加快台开快速路规划建设为切入点，重点推动台开同城，促进台山工业新城、开平翠山湖工业园和恩平（米仓-大槐）工业园的加快崛起，做大做强先进装备制造业。

　　二、经济蓄势

　　着力推动制造大市向制造强市转变。

　　落实珠西行动。全市要把珠西行动摆到经济工作举足轻重的战略位置加以推动，积极培育新的经济增长极。以发展先进（装备）制造业为主抓手，全面落实江门珠西行动工作纲要及五大行动[14]计划，积蓄发展能量，为经济起势打下坚实基础。以轨道交通装备、重型载重汽车及零部件、摩托车及零部件、光通信和视像装备、物流运输设备为重点，发挥广东南车、富华重工、大长江、海信、中集等龙头企业的引领作用。实行“六个优先”[15]，激励引导工业企业开展新一轮技术改造，完成技改投资65亿元、增长30%以上。

　　扩大产业投资。狠抓实体经济发展，紧盯项目产出率，着力推动138项、年度投资331亿元的产业重点项目建设，重点落实33个年度投资超亿元项目。建立市、县两级高效招商协作机制，组建专业招商队伍，开展精准招商、服务招商、环境招商。抓好高端新型电子信息、生物医药、绿色光源、新能源、新材料等战略性新兴产业发展，推动康师傅、李锦记（无限极）、天地壹号、嘉士利、维达、亚太纸业等食品、造纸板块做强做大，加快推动传统产业转型升级，实施智能化改造。支持现有企业增资扩产，发挥其建设周期短、产能见效快、用地制约少的“吹糠见米”效应。

　　推进创新驱动。鼓励创新创造，开展创新型城市建设和国家知识产权试点城市创建工作，以江门高新区和蓬江区为示范带动，引进一批创新型人才（团队），培育一批创新型企业，建设一批新型研发机构，打造一批新型产业社区，激发大众创业、万众创新新动能。推进市风投基金首期运作，年内成立第二期基金。完善国家半导体光电产品检测重点实验室、广东工业设计城江门基地等建设，培育广东省智能制造示范基地。组织实施100项市级以上重点科技计划项目，新培育35家国家级和市级高新技术企业，开展产学研项目80项，创建10家市级以上企业工程技术研究中心。加快金融、物流、工业设计等生产性服务业发展。深化全国小微企业信用体系试验区建设，加强金融第三方信用评级工作，推动江门粤澳中小微企业金融服务创新试验区建设。支持互联网经济发展，加强对电子商务运营服务企业引进、培育。大力扶持养老服务、健康产业发展。

　　主抓“一区六园”[16]。对园区平台实行统筹整合、优化调整、错位发展。严格实行节约集约用地，开展“七个一”专项行动[17]，加大对批而未供、供而未用及低效土地整理力度，建立新增建设用地指标分配与盘活存量建设用地挂钩机制，优先保障制造业项目用地。创新江门高新区体制机制，加快科技创新创业园、总部科技园等建设，做好第二期发债。加快发展海洋经济，依规推进广海湾填海工程，完成集中集约用海规划编制，珍惜利用岸线资源，推进“海域使用直通车”试点工作。

　　优化营商环境。充分发挥各级总商会（工商联）、行业商协会作用，开展企业家培训，推动“邑商再出发”。提供“一门式”便捷企业服务，收费就低不就高、办事就简不就繁、服务就近不就远，特别是对民营经济和中小微企业要高看一眼、多扶一把。探索建立民营融资服务联盟和民营企业“体检中心”[18]。推进多层次资本市场建设，争取今年1－2家企业在境内外上市，10家企业在新三板或股权交易中心挂牌，全力打造“江门板块”。加快外贸转型升级，推动新业态发展，继续推广关检合作“三个一”[19]区域通关一体化改革，稳定提升进出口规模。

　　着力推动旅游大市向旅游强市转变。编制全域旅游发展规划和实施方案。围绕探索建设国家级旅游产业示范区，打造粤港澳国际旅游休闲目的地，重点以西部旅游联盟为突破，台山重点探索全域旅游，推进滨海和川岛旅游规划编制，年内实现“川岛游一票通”，建设“广东第一步行径”；开平重点发展碉楼及风情古镇旅游，加快赤坎古镇项目建设，开平碉楼与村落力争成功创建国家5A级旅游景区，形成“广东旅游看碉楼”的品牌；恩平重点发展温泉旅游和航空题材；多形式多渠道引导、鼓励建设魅力小镇、生态乡村及“农家乐”示范点。推动东部旅游景点升级改造，华博馆、新会陈皮村、大雁山加快创建国家4A级景区。力争全市旅游总收入增长15%以上，突破300亿元。

　　着力推动农业大市向农业强市转变。制定实施建设现代农业强市工作纲要（2015－2020年）及行动方案。发展多种形式适度规模经营，新增市级以上农业龙头企业5家，市级示范家庭农场10家，农民专业合作社发展到1500家。完善和扩大农业“政银保”项目。加快开展农村综合改革试点工作，深化农村集体“三资”[20]管理，稳妥推进农村土地承包经营权确权登记工作。加大科技和金融支农力度，大力发展现代种业，提升园艺、冬种和禽畜产业规模化水平。发展农产品现代流通业和农产品加工业，开展“集中屠宰、冷链配送、生鲜上市”工作。加快发展“三品一标一名牌”[21]农产品。加强农村基础设施建设，全面完成29.9万亩和启动新建25.5万亩高标准基本农田建设任务，抓好各级水利设施工程建设。加快推动气象现代化。推进现代渔港建设。

　　三、城市振兴

　　启动新一轮规划修编。编制好“十三五”规划。以珠三角全域规划编制为契机，抓好各类总规、重要区域规划、综合交通、产业布局、公共服务等规划编制，以规划引领产城振兴。

　　启动交通大会战。把江门大道作为“一号工程”，按照2018年实现全线（鹤山桃源-新会崖门）通车的目标，力争江门大道蓬江段（五洞-龙湾立交）年底前建成，实现江顺大桥竣工通车。加快广中江、江罗、新台南延线等高速路建设，启动中开、鹤开高速建设，推进佛开高速三堡至水口段扩建前期工作。围绕打造互联互通、快进快出的高快速路网，启动台开快速路前期工作，加快推进325国道鹤山段、篁庄大道西延线、杜阮北路、迎宾西路、冲恩线等改造建设。加快江门高新区公共码头首期项目建设。推进深茂铁路江门段建设和广佛江珠城轨等前期工作。围绕打造全市主要铁路客站和对外交通门户、珠中江都市区地区性铁路综合交通枢纽、珠西与粤西城际铁路网核心枢纽、大型高铁客运站的目标，加快推动江门枢纽站场规划建设。抓好停车场的规划建设。

　　启动公园城市大行动。按照“统一规划、统筹开发、分步实施，属地建设、属地管理”的原则，研究制定全市公园城市建设工作纲要及行动方案，实施公园化战略，推进大地园林化，城市建公园，公园建城市，构建市域公园体系。坚持“抓两头，促中间”，一头抓龙头公园建设，东部主要抓圭峰山、大雁山、龙舟山（婆髻山、席帽山）、白水带，圭峰山着力打造“冈州第一峰”南粤文化名山，西部主要抓石花山、梁金山、鳌峰山（大人山）。另一头抓社区公园建设，今年新建、改建村（社区）公园330个，分期分批逐步推动全市所有村（社区）公园全覆盖。提升镇（街）公园建设水平。做好江门大道等主干道沿线绿化景观建设，继续深入开展种树绿化活动，年内种树250万株。启动慢行系统建设，推动沿江、沿河带状公园建设。有序推进全市饮用水源水库第一重山转为生态林计划。推进江门市植物园规划建设。抓好市属国有林场改革，组建林业集团。

　　启动新一轮环境大整治。严格执行新《环保法》，严格实行环保准入。探索成立专门打击食品药品安全和环境保护违法犯罪行为的执法机构，加大执法监管力度。推进市场化节能减排机制，实行能效“领跑者”制度[22]，完成“十二五”节能减排刚性指标。探索生态控制线划定工作。加大潭江流域水环境综合整治力度，推行“河长制”[23]，明确全市禁养限养区，扎实推进农业面源污染治理。加快建设恩平污水厂二期、开平迳头污水处理厂二期和杜阮、棠下污水处理厂再生水利用工程，完善污水管网，全市城镇污水处理率达到90%，污水处理再生水利用率超过10%。强化饮用水源保护，加快推进应急备用水源建设。加大以PM2.5、工业粉尘和城市扬尘为重点的大气污染防治力度，重点控制工业燃煤锅炉污染，基本淘汰“黄标车”，开展新能源汽车推广应用。向社会公开空气质量和主要河涌水质等环保信息监测情况。抓好城镇生活垃圾无害化处理和管道燃气设施规划建设。大力抓好农村环境卫生综合整治和宜居城乡创建工作，村庄整治覆盖全市80%以上的行政村。推进重点片区“三旧”[24]改造工作。

　　启动“中国侨都”建设。擦亮“中国侨都”的城市名片，打好“侨”牌，组建全市侨务工作协调小组，制定和启动全市侨务工作规划及行动方案。办好华侨华人博物馆和侨乡文化研究中心，加强外事、港澳工作和华侨新生代工作，支持“侨”题材文艺作品创作，推动将侨乡文化作为学校教学内容。加强对华侨建筑历史文化街区和文物的保护，推进我市海上丝绸之路史迹点申遗，做好北京新会会馆保护复建工作。

　　四、民生保障

　　加大民生投入，全市安排民生支出120.7亿元，同比增长12%。进一步完善创文长效机制，深化“大民政”工作，继续推进基本公共服务均等化综合改革工作，抓好十件民生实事（见附件3）落实，让广大市民群众得到更多实惠。

　　精准扶贫。统筹帮扶城乡贫困人群[25]，实施精准脱贫，充分发动社会力量积极参与，努力实现扶贫全覆盖、常态化。试行资源激励型财政奖励政策，鼓励农村积极保护基本农田、公益林和水源地等自然资源，力争年内全面消灭贫困村，使全部村委会集体收入达到10万元以上。东部城镇低保月人均补差达到384元以上，农村达到186元以上；西部城镇低保月人均补差达到374元以上，农村达到172元以上。确保五保供养标准达到上年度农村居民人均纯收入的65%以上。全面建立临时救助制度。实现低保残疾人生活津贴和重度残疾人护理补贴分别提高到1200元／年和1800元／年。进一步提高孤儿供养水平。完成棚户区改造和保障性住房建设2027套。

　　服务便民。实行“一个平台全覆盖”，在全市所有村（居）建立统一的社区公共服务站，落实103项基本公共服务项目，努力实现服务就在“家门口”。提高公共文化服务水平，深入开展文化下乡、进社区、进校园活动。继续深化社区警务建设，推动便民业务前移到基层所队、社区警务室或社区公共服务站办理。

　　公交一体。启动实施东部公交一体工作方案，优化调整公交线网，按照“设施一体、标识一体、换乘一体、服务一体”的标准打造公交系统，新增公交车120标台[26]，建设改造100个候车亭，推进东部公交互联互通，为推进西部及全市公交互联互通打下基础。落实产业园区公交首末站、枢纽站、主要旅游点和公园等公共场所上落站的用地和规划建设。积极改善农村交通条件，完成150公里农村公路建设。

　　就业保障。实施精准化就业帮扶，全面开展“失业保险一站式”服务。加强本地紧缺专业人才培养，鼓励企业充分用好本地劳动力资源。全市城镇新增就业4.5万人以上，城镇失业人员再就业3万人，应届高校毕业生就业率达90%以上。进一步提高社会保险各项待遇，完善全征地农民养老保障政策和社会保险基金预警机制，稳步推进机关事业单位养老制度改革。

　　以文化人。制定和启动2015-2020年文化强市规划及行动方案。加快推进“四馆”[27]整合提升，年底前完成文化馆和美术馆改造，启动图书馆改造。三区四市公共图书馆、文化馆达到国家一级馆标准，镇（街）综合文化站80%达到省一级站标准。完善“10分钟体育健身圈”设施布局，加快江门体育中心、江门演艺中心建设，打响“四个一”[28]活动品牌，打造“中国舞蹈之城”。加快工艺美术、印刷发行、动漫等文化产业发展。

　 教育优先。巩固城乡义务教育均衡发展，完善教育布局规划，改善义务教育阶段学位不足状况，规划新建扩建一批中小学校，鼓励扶持优质民办教育，推动教育优质均衡发展。年内基本建立普惠性学前教育体系，做好台山、开平、鹤山申报广东省推进教育现代化先进市（区）工作。巩固提升高等教育发展水平。积极推进校企深度合作，推动校企共建生产性实训基地，建设中等职业教育改革发展示范学校。抓好老干部（老年）大学、社区大学和特殊教育学校的改造建设。

　　健康惠民。稳步推进县级公立医院综合改革，进一步优化整合市、县两级医疗资源配置。鼓励发展优质民营医疗机构，引导社会养老机构与医疗机构合作。整合镇级计生服务机构与卫生院职能，基本实现农村卫生站全覆盖，提高基层卫生服务水平。建立完善医患纠纷第三方协调处理机制。推进广东银葵医院建设。启动医疗废物处理中心规划建设。确保完成省下达的人口计生任务。

　　平安江门。牢固树立安全生产的红线意识和底线思维，强化安全生产基层基础建设和企业主体责任落实，坚决遏制较大以上事故发生。着力推进大巡防工作，加强公安指挥中心平台建设。做好综治信访维稳工作。完善公共突发事件应急处置体系建设，提高应急管理能力。

　　此外，要进一步完善国防动员体系，落实双拥和优抚安置工作。提高城市民族工作水平。继续做好宗教、对台、人防、打私、统计、档案、方志、妇女儿童、红十字会等工作。

　　五、政府提效

　　建设法治政府。法治政府是依法治市的重中之重。必须坚持依法行政，加快建设职能科学、权责法定、执法严明、公开公正、廉洁高效、守法诚信的法治政府。主动接受人大监督，认真执行市人大及其常委会各项决议决定，支持政协委员参政议政，提高人大代表议案、建议和政协提案办理水平。建立健全直联制度。继续发挥市决咨委的作用，探索成立“江门智库”。设立政府法律顾问室，推行法律顾问制度。深化商事制度改革，强化商事登记后续监管。严格落实党风廉政建设责任制，严格执行中央“八项规定”。按照省的部署完成公务用车制度改革。做好政府预算及“三公”经费公开，“三公”经费和一般性工作经费支出均压减10%，实现财政供养人员只减不增。抓好政府性资金和投资项目审计监督工作。

　　建设透明政府。坚持“市场事，市场定；社会事，社会建；政府事，公平办”，制定公布市、县两级政府权责清单，厘清市县镇村权责利关系，市一级重在统筹、监管与协同，县一级和重点产业园区是经济发展的主战场，镇（街）是社会治理的主平台，村（社区）是社会公共服务的末梢与终端。政府行政全面实行“清单管理、阳光行政、社会监督”的运行机制，全面公布“1+3”清单[29]，探索市场准入负面清单，坚持“非禁即可”，鼓励支持社会资本进入公共事业建设领域，继续推进国有资产整合运营，积极发展混合型经济，探索PPP合作模式[30]项目；列明行政审批清单，全面压减行政审批自由裁量权，公布涉企收费目录清单；制定政府监管清单，严格规范公正文明执法，建立健全行政执法案卷评查制度，探索执法公开制度；力求做到禁限清晰、准许标准、监管有力。着力加强政务网站、微博群和微信公众号建设，实行市县镇三级政务微博全覆盖，完善政府新闻发布制度，扩大公众知晓权和参与权。

　　建设诚信政府。提高政府公信力是“信用江门”建设的立足点和着力点。要抓好诚信政府建设试点工作。大力整治机关工作人员行政“不作为、慢作为、乱作为”现象，严肃查处“吃拿卡要”行为，营造按规矩办事、按规矩用权的政务环境。建立黑名单制度，全面构建社会信用体系。完善提升集政府采购、工程招投标、土地招拍挂、产权交易、物业招租、集体经济交易等功能于一体的市公共资源交易中心建设水平，实现公共资源交易标准化、公平化、规范化。完善投融还体制建设，健全投融资管理机构，确保重大基础设施项目建设资金及时到位、规范使用，将政府债务纳入预算管理，强化风险防范。

　　建设效率政府。加强队伍建设，着力提升政府执行力。启动智慧江门建设，建立“一张网”、“一块屏”、“一点通”的统一的政务服务系统。探索推动全市数据统筹机构建设工作，构建全市政务信息数据服务平台。推进办公自动化系统应用和升级，实现市本级与三区四市电子公文交换系统互联互通。做好商事主体信息管理和公示平台、联合征信系统、电子政务云计算中心、地理信息共享平台等一批政务信息化重点项目的建设和完善。加强便民服务平台建设，提高政府服务热线“智能化”建设水平，以大数据服务大民生，推进行政服务标准化、便民化。进一步激发社会组织活力，规范社会组织，发展壮大社工人才队伍，继续推动社区网格化管理试点工作。启动城市建设档案馆规划建设。推进行政服务中心、应急指挥中心资源整合，建立健全对重大突发事件的快速反应和应急机制。

　　各位代表，同志们，今年的政府工作特别繁重，各级各部门必须勇于负责、勇于担当，善于谋、敢于拼，沉下心来谋实事，沉下身子抓落实，让各项决策落地，让各项举措惠民，努力交出一份得到各位代表和广大市民群众点赞的答卷。
